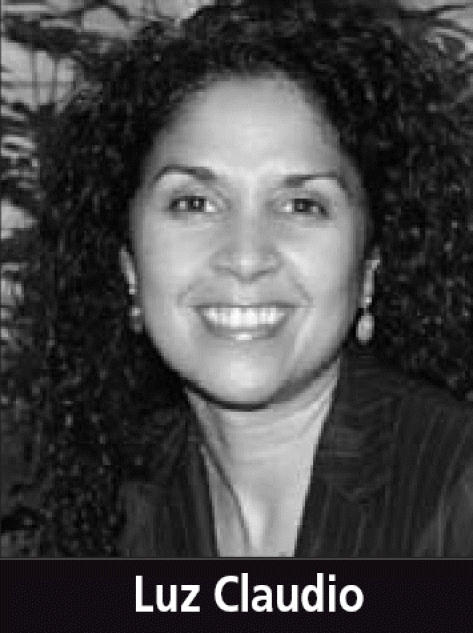# Guest Editorial: Global Perspective on Environmental Health

**DOI:** 10.1289/ehp.114-1551994

**Published:** 2006-08

**Authors:** Roger I. Glass, Kenneth Bridbord, Joshua Rosenthal, Luz Claudio

**Affiliations:** Fogarty International Center, National Institutes of Health, Bethesda, Maryland, E-mail: glassr@mail.nih.gov; Mt. Sinai School of Medicine, New York, New York

The environment is a key determinant of human health, and exposures to toxic chemicals, physical factors, and pollutants all have a direct impact on the quality of life, the burden of disease, and the outcome of longevity. In the developing world, population growth with urban crowding, the introduction of many environmental pollutants and toxic exposures, and the lack of clear policies to control pollution have accentuated the negative impact that these environmental factors can have as causative factors of disease in humans. Research to address environmental problems in any country, rich or poor, can lead to greater understanding of the pathogenesis of disease processes caused by untoward environmental exposures and can guide research to improve diagnosis, treatment, and control. In fact, targeting the best scientific research to environmental problems, wherever they arise, can lead to breakthroughs in our understanding that can be of great benefit to all.

In the developing world, such problems of environmental health can be elusive, widespread, and disguised and sometimes appear where least expected. In the 1970s and 1980s, one of us (R.I.G.) worked in Bangladesh and followed a program to install tube wells in many rural communities in an effort to control the annual outbreaks of cholera that were believed to be spread by contaminated river water. This program, which was admirable in its intent, had a tragic outcome that could never have been anticipated. Two decades after the program was completed, high levels of arsenic were identified in water from these tubewells that had left a huge population with chronic exposure to toxic levels of arsenic. Identification of arsenic in this water has led to a national—and indeed international—effort to understand the extent of the problem, to assess the health impact of chronic arsenic poisoning in this population, to test novel methods of treatment, to seek the environmental source of the problem, and to design control programs to diminish this unforeseen hazard. By applying quality science to this investigation, we can learn a great deal about how to diagnose chronic arsenosis earlier, understand its pathogenesis and long-term sequelae, identify more effective treatments, develop simple laboratory methods to screen water samples, and test different public health methods for prevention. The problem might well have arisen anywhere, but the opportunity to study this problem and intervene is clearly at the center of environmental health in a global arena.

A similar and complex problem has been observed with indoor air pollution, particularly in the developing world. One key indicator of the health of a society is the measure of mortality among children < 5 years of age. The most common cause of death in this age group is acute respiratory disease, a syndrome usually linked to a wide variety of infectious agents and asthma. However, a key underlying condition that places these children at particularly high risk of death is indoor air pollution from cooking and heating fires in the home. For research to be able to improve the health and long-term outcome for these children, the following questions need to be answered: What are the etiologic agents involved? Which ones are affected by in-home pollution? What measures can be introduced to diminish the risk to children? And can these interventions decrease childhood mortality? Research could further our understanding of the entire disease process, the interaction of air pollution on immunity and infection, and the public health measures needed to improve air quality in a home heated by an open fire. Again, multidisciplinary research involving clinicians, toxicologists, immunologists, microbiologists, epidemiologists, and public health specialists is key to addressing these complex problems. The benefits of this research could have global implications for improving child health both at home and abroad.

What is needed to address these important problems of environmental health in the developing world? How can we begin to identify particularly hazardous exposures and bring the best science to bear in understanding the problems and seeking culturally appropriate solutions? How could the United States benefit from supporting research on these toxic exposures and environmental hazards overseas? The Fogarty International Center has been partnering with the National Institute of Environmental Health Sciences (NIEHS) on a series of programs to expand the capacity of investigators in developing countries to take on this mission and develop their own research agendas in environmental health. For example, the International Training and Research Program in Environmental and Occupational Health (ITREOH) was established to enable programs at universities and nonprofit research institutions in the United States to train researchers from the developing world and support their research activities when they return home. The program is building global capacity and collaborations to better identify, investigate, understand, prevent, and control occupational and environmental problems where they occur. At its inception in the mid-1990s, the program focused on surveillance and the assessment of risk. As it has evolved, the focus has moved toward prevention and control. Along the way, we have come to understand some of the challenges that can yield their answers through a program of basic and applied research. By focusing on major environmental problems, working through major centers of academic excellence, and identifying investigators early in their careers who can be trained to the task, the program will build the next generation of leaders to continue these efforts and enhance the ability of local institutions to become centers of excellence linked to well-established collaborators in academic centers throughout the United States.

As we enter the 21st century, the problems caused by environmental hazards are multiplying and becoming more visible due to rapid population growth, crowding, and industrialization and pollution from many sources. The field of environmental health, like medical research in general, is profiting from new tools to detect hazardous exposures more rapidly; research their causes; understand genetic, physiologic, and immunologic modifiers of risk; and seek novel means of treatment, understanding, and control. The recent identification in India that smoking (and perhaps indoor air pollution) might be a key determinant of death from tuberculosis demonstrates that environmental hazards can play an often hidden but critical role in human health. It will only be through international research collaborations, training of and support for the next generation of researchers in the developing world, and involvement of multidisciplinary research teams that we can hope to attack the complex problems of environmental health. Maintaining an international focus ensures that we can recruit the largest group of scientists to this effort, identify areas and problems posing the greatest hazards to human health, and seek the most rapid resolution through collaborative networks of quality research. Such efforts, already begun, and collaborations between groups with similar goals, such as the NIEHS and the Fogarty International Center, could help build a constituency to identify and diminish the many risks posed by environmental hazards.

The challenge before us in the international arena of environmental health is great and expanding, and failure to appreciate the extent of the problem could have adverse consequences for us all. Much can be addressed by developing centers of excellence in academic centers in the developing world and training local staff so that they are capable of researching key issues of environmental health and establishing collaborations. The best science can be used to improve our understanding of the problems of global environmental health and their resolution.

## Figures and Tables

**Figure:**
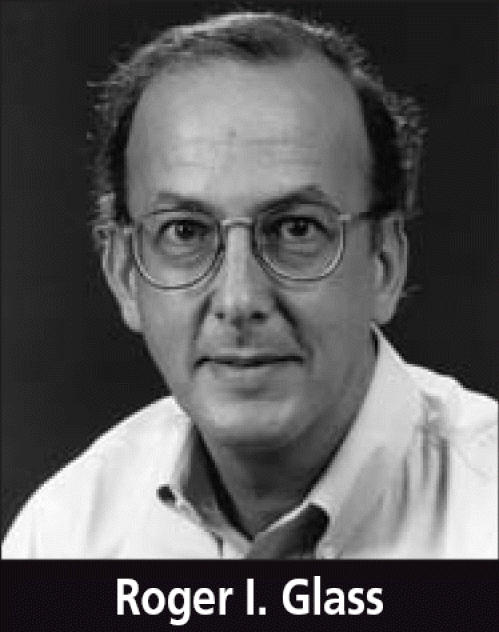


**Figure:**
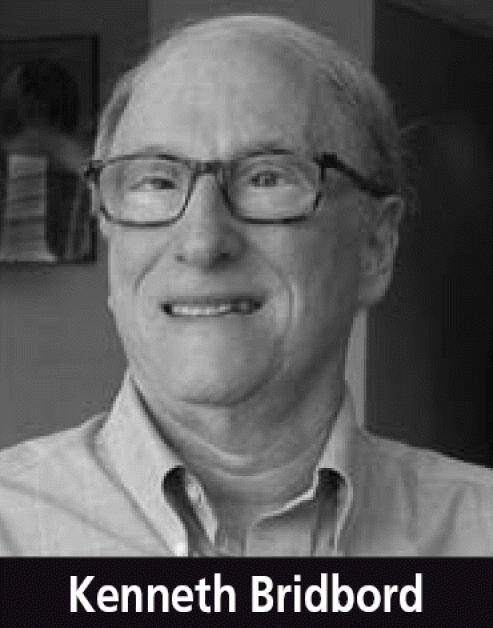


**Figure:**